# Multiple Intravitreal Ranibizumab Injections for Persistant Choroidal Neovascularization Associated with Presumed Ocular Histoplasmosis Syndrome

**DOI:** 10.4274/tjo.82956

**Published:** 2017-04-01

**Authors:** Turgut Yılmaz, Seyhan Dikci, Oğuzhan Genç, Kayhan Mutlu

**Affiliations:** 1 İnönü University Turgut Özal Medical Center, Department of Ophthalmology, Malatya, Turkey

**Keywords:** choroidal neovascularization, histoplasmosis, intravitreal injection, ranibizumab

## Abstract

Presumed ocular histoplasmosis syndrome (POHS) is a clinical entity that is characterized by small, round, discrete, macular or mid peripheral atrophic (punched out) chorioretinal lesions (histo spots), peripapillary scarring, choroidal neovascularization (CNV), and the absence of anterior uveitis and vitritis. Diagnosis of this disorder is based upon characteristic clinical findings and a positive histoplasmin skin test or residence in an endemic region for *Histoplasma capsulatum*. There is no active systemic disease during diagnosis of POHS. Disciform scarring and macular CNV secondary to POHS is a well-known complication which leads to loss of visual acuity or visual disturbance. Without therapy, the visual prognosis in these patients is unfavorable. Submacular surgery, radiation, steroids, photodynamic therapy, and most recently anti-vascular endothelial growth factor therapy are current therapeutic options for this condition. We report a case with persistent CNV secondary to POHS in a middle-aged woman with moderate myopia and the clinical course of treatment with multiple intravitreal ranibizumab (Lucentis^®^, Novartis) injections.

## INTRODUCTION

Presumed ocular histoplasmosis syndrome (POHS) was first described in 1959 by Woods and Wahlen^1^ as peripheral chorioretinal scar and hemorrhagic macular disciform lesion in a patient with positive histoplasmin skin test. Today, POHS is accepted as an entity characterized by small, round, discrete, atrophic mid-peripheral chorioretinal lesions (called histo spots), peripapillary scarring, and choroidal neovascularization (CNV).^2^ These patients do not exhibit anterior segment or vitreous inflammation. Diagnosis is based on typical clinical findings, as well as positive histoplasmin skin test or history of exposure to the pathogen such as residence in an endemic area for *Histoplasma capsulatum*.^3^ The histoplasmin skin test becomes positive soon after exposure and remains positive throughout life. However, this test is not used in contemporary practice due to the possibility (albeit unproven) that the histoplasmin skin test can reactivate latent ocular disease.^3^ Although *H. capsulatum* can rarely be isolated or cultured in patients suspected of having POHS, patients with typical clinical findings are considered to have this fungal infection.^4^

The main causes of vision loss associated with POHS are macular CNV and formation of disciform scar. Without treatment, the prognosis for these patients is poor.^2^ Treatment should aim to minimize the area of scarring and thus reduce the size of scotoma. In this report, we present a case with persistent CNV secondary to POHS in a myopic, middle-aged woman treated with multiple intravitreal ranibizumab (Lucentis^®^, Novartis) injections.

## CASE REPORT

**Figure 1:**
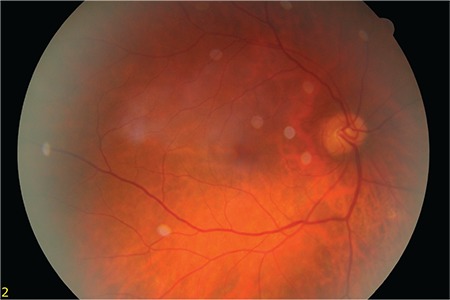
Tilted optic disc and tigroid fundus in the right eye

**Figure 2:**
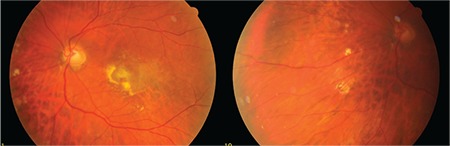
Lesions consistent with macular choroidal neovascularization and a few small, discrete mid-peripheral chorioretinal scars in the left eye

**Figure 3:**
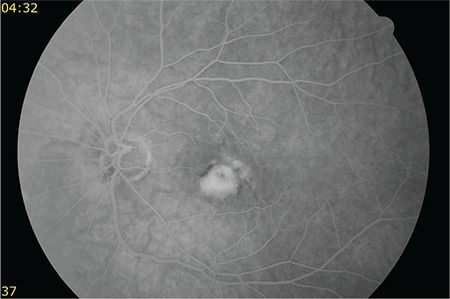
Fundus fluorescein angiography shows juxtafoveal leakage consistent with classic choroidal neovascularization in the left eye

**Figure 4:**
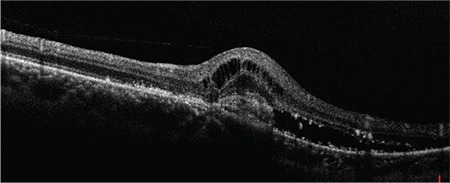
Optical coherence tomography shows intraretinal and subretinal fluid due to choroidal neovascularization in the left eye

**Figure 5:**
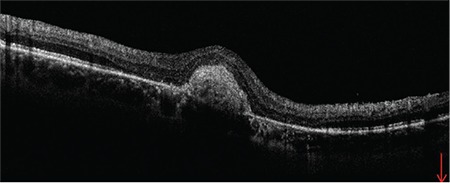
Optical coherence tomography shows the subretinal scar post-treatment

A 50-year-old female patient presented to our clinic with vision loss in her left eye that she noticed 3 months earlier. She had refractive errors of -3.50 (-1.25x135) in the right eye and -4.00 (-1.00x180) in the left eye. Her best corrected visual acuity (BCVA) was 1.0 in the right eye and 0.6 in the left eye. Anterior segment examination and intraocular pressure measured by Goldmann applanation tonometry were normal in both eyes. Fundus examination with 90-D lens revealed peripapillary atrophy and peripheral tigroid fundus in both eyes. Furthermore, tilted optic disc was observed in the right eye, while lesions consistent with macular CNV and a few small, discrete mid-peripheral chorioretinal scars were observed in the left eye (Figures 1 and 2). Fundus fluorescein angiography examination revealed juxtafoveal leakage consistent with classic CNV in the left eye (Figure 3). Optical coherence tomography revealed intraretinal and subretinal fluid due to CNV in the left eye (Figure 4). A detailed history was obtained and it was learned that the patient had a pet bird. She was diagnosed with POHS based on typical clinical findings. Intravitreal ranibizumab therapy was recommended to treat the CNV. During 1.5 years of follow-up, a total of 5 ranibizumab (Lucentis^®^, Novartis) injections were applied, with the first 3 administered once a month over the first 3 months. After the injections, the CNV regressed leaving a subretinal scar, and the patient’s BCVA in the left eye remained at 0.3 (Figure 5).

## DISCUSSION

*Histoplasma capsulatum* is a dimorphic fungus generally carried by birds and bats. It is distributed worldwide and is endemic in some regions, such as the USA.^5^ Ocular symptoms usually emerge years after exposure to the agent. There are no specific anterior or posterior segment inflammation signs specific to POHS that distinguish it from other retinopathies.^6^ Multifocal choroiditis (MFC) is among a group of diseases called primary inflammatory choriocapillaropathies whose pathology involves choriocapillaris perfusion abnormalities.^7^ POHS can be difficult to differentiate from MFC because their clinical and FA findings are similar. As in MFC, inflammatory CNV leading to vision loss or visual disturbances is also common in POHS.^7^ However, while anterior segment and vitreous inflammation are evident in MFC, this sign is absent in POHS. Furthermore, punctate inner choroidopathy, pathologic myopia, sarcoidosis, tuberculosis, and other causes of chorioretinitis may be considered.^3^

POHS-associated CNV generally occurs in the second and fifth decades and often develops as classic CNV.^8^ The disease remains asymptomatic until the macula is affected. Central vision loss is generally seen in the affected eye due to scar formation after serous or hemorrhagic detachment related to CNV. It is estimated that 5% of POHS patients will develop disciform lesions, and there are findings indicating that these develop in areas of the fundus with previous atrophic scar.^4^ On that basis, the incidence of disciform lesion development is predicted to be 10 times higher in patients with perimacular atrophic scars compared to those without lesions in this region.^4^ Vision of 20/40 or better could be preserved in only 15% of patients with CNV involving the fovea. Compared to other causes of CNV, the rate of fellow eye involvement is lower at 1.5 to 2% per year.^4^ Visual prognosis is poor in POHS patients, and 20% of patients develop bilateral disciform macular disease.^4^ POHS is also one of the rarer causes of peripapillary CNV.9 Young age, good initial visual acuity, a relatively smaller CNV area, and lack of fellow eye involvement are criteria for good prognosis.^3^

Because the pathophysiology of POHS is not fully understood, current treatment approaches target CNV, which is a major cause of vision loss in these patients.^5^ These treatments include laser photocoagulation, photodynamic therapy (PDT), local and systemic corticosteroids, radiation, submacular surgery, macular translocation and, most recently, intravitreal anti-vascular endothelial growth factor (anti-VEGF) implantation.^2,4,5,10,11^ With the exception of anti-VEGF therapy, all of these approaches have potential drawbacks and high recurrence rates.^2^ Studies on the causes of inflammatory CNV have shown that VEGFs contribute to CNV development and progression in these types of cases.^4^ Although it is not known exactly what causes POHS-associated CNV, available data suggest that angiogenetic factors like VEGFs play an important role. As a result, anti-VEGF therapy appears to have an important role in the treatment of this disease.^4^ Case reports and case series support the efficacy of anti-VEGF agents in the treatment of CNV secondary to POHS.^4,12,13,14^

The efficacy of ranibizumab to treat CNV was investigated in a phase 1, randomized clinical study including a total of 30 patients (9 POHS patients) with CNV due to causes other than age-related macular degeneration and was reported to be effective in these patients.^12^ Nielsen et al.^13^ stated that anti-VEGF agents were beneficial in the treatment of CNV related to ocular histoplasmosis syndrome and found that these patients required an average of 4.5 intravitreal injections per year. Recently, Ramaiya et al.^14^ compared the efficacy and safety of PDT and intravitreal ranibizumab in the treatment of POHS-associated CNV and found that all patients in the PDT group required rescue ranibizumab injections and that the mean injection number was 7.7 in the ranibizumab group and 2.5 in the PDT group. None of the patients in either group had vision loss during the follow-up period; at 1-year follow-up, 80% of the ranibizumab group and 50% of the PDT group showed visual gains of 15 letters or more. Based on these results, Ramaiya et al.^14^ emphasized that ranibizumab alone or in combination with PDT may be effective treatment options for neovascular complications associated with POHS. None of the studies mentioned reported serious side effects related to treatment.^12,13,14^ In our case, CNV regressed after a total of 5 doses of intravitreal ranibizumab, leaving a subretinal scar.

## CONCLUSION

Turkey is not among the areas where Histoplasma capsulatum is endemic, and thus POHS is not a common clinical condition in Turkey. Intravitreal pharmacotherapy is widely used to treat CNV arising due to various causes. For these rare cases, practitioners should keep in mind that intravitreal ranibizumab injection is a safe and effective treatment method and that multiple anti-VEGF injections may be required.
